# Oxidative Stress State Is Associated with Left Ventricular Mechanics Changes, Measured by Speckle Tracking in Essential Hypertensive Patients

**DOI:** 10.1155/2015/502107

**Published:** 2015-10-04

**Authors:** Luis Antonio Moreno-Ruíz, David Ibarra-Quevedo, Erika Rodríguez-Martínez, Perla D. Maldonado, Benito Sarabia-Ortega, José Gustavo Hernández-Martínez, Beda Espinosa-Caleti, Beatriz Mendoza-Pérez, Selva Rivas-Arancibia

**Affiliations:** ^1^UMAE Hospital de Cardiología, Centro Médico Nacional Siglo XXI, IMSS, 06720 Mexico City, DF, Mexico; ^2^Departamento de Fisiología, Facultad de Medicina, UNAM, 04510 Mexico City, DF, Mexico; ^3^Instituto Nacional de Neurología y Neurocirugía, Manuel Velasco Suárez, 14269 Mexico City, DF, Mexico

## Abstract

The oxidative stress state is characterized by an increase in oxygen reactive species that overwhelms the antioxidant defense; we do not know if these pathological changes are correlated with alterations in left ventricular mechanics. The aim was correlating the oxidative stress state with the left ventricular global longitudinal strain (GLS) and the left ventricular end diastolic pressure (LVEDP). Twenty-five patients with essential hypertension and 25 controls paired by age and gender were studied. All of the participants were subjected to echocardiography and biochemical determination of oxidative stress markers. The hypertensive patients, compared with control subjects, had significantly (*p* < 0.05) higher levels of oxidized proteins (5.03 ± 1.05 versus 4.06 ± 0.63 nmol/mg), lower levels of extracellular superoxide dismutase (EC-SOD) activity (0.045 ± 0.02 versus 0.082 ± 0.02 U/mg), higher LVEDP (16.2 ± 4.5 versus 11.3 ± 1.6 mm Hg), and lower GLS (−12% versus −16%). Both groups had preserved ejection fraction and the results showed a positive correlation of oxidized proteins with GLS (*r* = 0.386, *p* = 0.006) and LVEDP (*r* = 0.389, *p* = 0.005); we also found a negative correlation of EC-SOD activity with GLS (*r* = −0.404, *p* = 0.004) and LVEDP (*r* = −0.347, *p* = 0.014).

## 1. Introduction 

An increasing body of evidence suggests that oxidative stress is involved in endothelial dysfunction, a factor that is present in the pathogenesis of many cardiovascular diseases, including hypercholesterolemia, atherosclerosis, hypertension, diabetes, and heart failure [1–4]; endothelial dysfunction is what all of these diseases have in common [5].

In healthy individuals, the endothelium plays a key role in vascular homeostasis through the release of a variety of antiatherogenic substances that exert an effect by way of platelet aggregation and adhesion inhibition, smooth muscle cell proliferation, and leukocyte adhesion [6–17].

The evidence that oxidative stress state occurs in hypertensive patients is based on increased levels of biomarkers such as oxidized protein and lipid oxidation and on the decreased activity of antioxidants such as superoxide dismutase, glutathione peroxidase, and catalase [18–20] as well as on the shorter telomere length found in these patients [21, 22]. Some studies have revealed an association between oxidative stress, inflammation, and arterial hypertension. These factors participate in a vicious cycle that can lead to and perpetuate progressive cardiovascular diseases [23]. It is now clear that oxidative stress has an important role in the impairment of cellular signal pathways and the modulation of growth, apoptosis, hypertrophy, inflammation, and remodeling of cardiac muscle [24] through the activation of various signaling cascades and redox state-sensitive transcription factors [25, 26]. ROS generates various stimuli, including angiotensin II, endothelin-1, cytokine, and growth factor-induced redox-sensitive signals, and also oxidative stress state produces the decrease of extracellular superoxide dismutase (EC-SOD) activity and increased oxidized proteins leading to the accumulation of extracellular matrix proteins, interstitial and perivascular fibrosis, and myocyte hypertrophy, which cause left ventricular (LV) remodeling as in the case of diabetic cardiomyopathy [27–29].

The evidence suggests that systolic and diastolic abnormalities in these patients begin to develop in the early stages of the disease, even when the left ventricular ejection fraction (LVEF) is still normal [30].

The ultrasound evaluation of myocardial deformation by 2-dimensional strain based on the speckle tracking of grayscale images enables the accurate quantitation of regional and global systolic function by determination of the longitudinal, circumferential, and radial deformation of myocardial fiber in the LV, providing information about structural and functional abnormalities [31, 32].

Conventional transmitral Doppler flow and assessment of mitral annular velocities by tissue Doppler allowed us to study the diastolic function by measurements of E velocity, E/E′ ratio and left ventricular end diastolic pressure (LVEDP) [33–35].

The aim of this study was to correlate the oxidative stress state (levels of oxidized proteins and EC-SOD activity) with LV mechanics assessed by left ventricular global longitudinal systolic strain (GLS) and left ventricular end diastolic pressure (LVEDP).

## 2. Materials and Methods 

### 2.1. Subjects

Subjects were adults of both genders with a previous diagnosis of essential hypertension, systolic pressure ≤150 mm Hg, and diastolic pressure ≤90 mm Hg. All hypertensive patients were on medication for blood pressure control. These patients were referred from a second level hospital to the echocardiography laboratory of the Hospital de Cardiología, CMNSXXI, IMSS. The eligibility criteria for this study included the following: ejection fraction ≥0.50, sinus rhythm, NYHA functional classes I-II, and absence of other systemic disease. Fifty patients were included in this study, who were divided into two groups: twenty-five patients in essential hypertensive group (mean age 43.3 ± 8.04 years), of which 40% were women and also 25 patients in the control group (mean age 43.8 ± 10.2 years) of which 40% were also women. Patients with bidimensional echocardiographic images of poor quality or not susceptible to data analysis in QLAB software were excluded (Phillips). For control, we selected healthy subjects, paired with the hypertensive patients by gender and age. The protocol was carried out in strict accordance with ethical norms, the Declaration of Helsinki, and the regulations of the General Health Law on health research and approved by the local research committee and ethics board. All participants signed a written informed consent. All subjects refrained from eating or drinking anything for 8 to 12 hours before each of the testing sessions. Demographic data and anthropometric measurements were collected. Finally, echocardiographic images and blood samples were taken from the antecubital vein in a 7 mL tube (BD Vacutainer EDTA) at room temperature (14–24°C), which was then processed by centrifugation.

### 2.2. Echocardiographic Study

**Figure 1:**
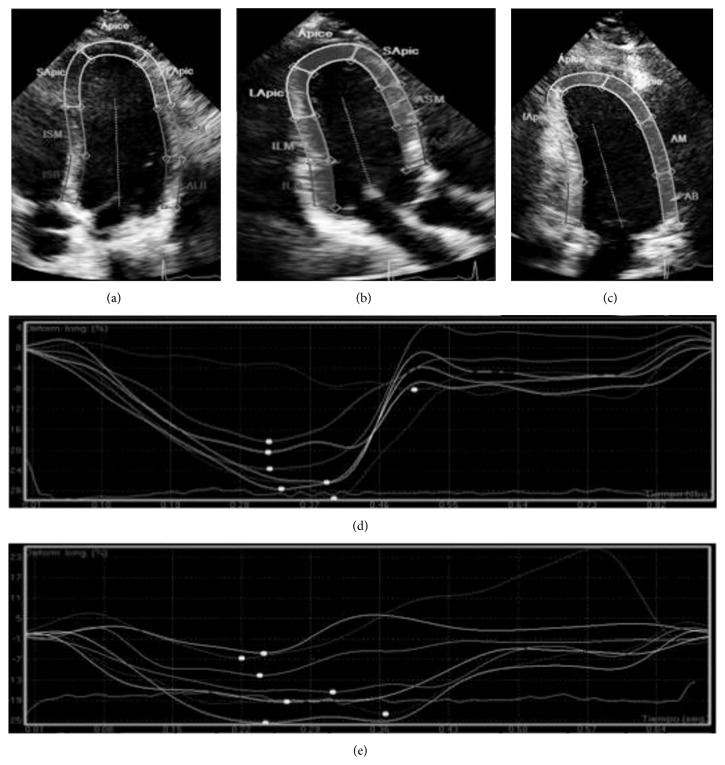
Echocardiographic determination of myocardial longitudinal deformation. Selection of regions of interest for longitudinal strain quantification in four chambers (a), three chambers (b), and two chamber (c) projections. Longitudinal strain curves of a control (d) and hypertensive (e) patient.

Two-dimensional echocardiography was performed and images were taken from patients in left lateral decubitus position using a commercially available system (Phillips iE33). Image acquisition was performed using an S5-1 transducer. The images were taken at a depth of 16 cm using standard parasternal and apical views (standard long-axis and 2-, 3-, and 4-chamber images) with a frame rate of 80–100 frames/s. Standard M-mode and 2D images were acquired in cine loop format, including pulsed, continuous, color, and tissue Doppler data from three consecutive heart beats. All analyses were performed offline using commercial software (QLAB, Phillips). Myocardial deformation measurements were performed using speckle tracking. A global longitudinal strain curve was obtained (Figures 1(d) and 1(e)) from each of the apical views (Figures 1(a), 1(b), and 1(c)); all LV myocardial segments were considered to be the region of interest. The average value of each peak systolic longitudinal strain from the three apical views was calculated as GLS and expressed as percentage. Estimated LVEDP values were calculated using Nagueh formula, 1.9 + (1.24 × E/E′), and expressed as mm Hg [36].

### 2.3. Biochemical Analyses

Blood samples were collected from the antecubital vein in two 7 mL tubes (Vacutainer EDTA) for biochemical analysis. Plasma samples were obtained by centrifugation at 100 g for 10 minutes. Plasma was separated into aliquots and frozen at −30°C until assays were performed. The content of protein carbonyl in the samples was determined by the method of Reznick and Packer modified for plasma proteins [37]. Carbonyl formation was assessed on the basis of the formation of protein hydrazone by reacting with 2,4-dinitrophenylhydrazine (DNPH). Plasma aliquots were incubated overnight in streptomycin sulfate 10% to remove nucleic acids and centrifuged at 21000 g at 48°C for 40 min. The samples were then treated with 10 mM DNPH (in HCl 2.5 M) for 1 h at room temperature; afterwards, trichloroacetic acid 10% was added and the samples were centrifuged at 2500 g at 48°C for 10 min. The pellets were washed three times with ethanol/ethyl acetate (1 : 1), dissolved in 6 M guanidine hydrochloride (phosphate buffer 20 mM, pH 7.4), incubated for 10 min at 37°C, and centrifuged at 5000 g and 48°C for 3 min to remove insoluble material. Absorbance was measured at 370 nm. Protein carbonyl content was expressed as nmol carbonyl/mg protein using the molar absorption coefficient of DNPH (22000 M^−1^ cm^−1^). Total protein concentration was obtained by reading the optical density at 280 nm in blank tubes prepared in parallel (treated only with HCl). The standard curve of bovine serum albumin (0.25–2 mg/mL) prepared in 6 M guanidine-HCl was used for this analysis.

Extracellular superoxide dismutase (EC-SOD) activity in plasma samples was determined by the method of Oberley and Spitz [38]. In brief, competitive inhibition assays were performed using the xanthine oxidase system to reduce Nitro-Blue Tetrazolium (NBT). The mixture contained a final concentration of EDTA 0.122 mM, NBT 30.6 *μ*M, xanthine 0.122 mM, bovine serum albumin 0.006%, and sodium carbonate 49 mM. Five hundred *μ*L of homogenates (diluted 1 : 20) was added to 2.45 mL of the mixture described above. Afterwards, 50 *μ*L of xanthine oxidase was added in a final concentration of 2.8 U/L and the mixture was incubated in a water bath at 27°C for 30 min. The reaction was stopped with 1 mL of 0.8 mM cupric chloride and the optical density was read at 560 nm. One hundred percent of NBT reduction was obtained in a different tube in which the sample was replaced by distilled water. The amount of protein involved in inhibiting the reduction of NBT to 50% of the maximum was defined as one unit of SOD activity. The results were expressed as U/mg protein.

### 2.4. Statistical Analysis

The data were analyzed with IBM SPSS statistical software 21.0. Quantitative variables were expressed as means ± SD or median (interquartile range) according to their distribution. Comparisons between groups were performed using Student's *t*-test or Mann Whitney *U* test. Pearson's linear correlation was used to determine whether there is an association between biomarkers of oxidative stress and global LV longitudinal strain or LV telediastolic pressure. Multivariate analysis was used. A value of *p* < 0.05 was considered significant.

## 3. Results 

Twenty-five healthy subjects (age 43.3 ± 8 years) and 25 patients with hypertension (aged 43.8 ± 10.2 years) were two groups, both groups with similar ages. Anthropometric and clinical variables are presented in Table 1. We detected a higher prevalence of overweight in the essential hypertension group evaluated by body mass index (24.8 ± 1.0 versus 29.4 ± 1.8, *p* < 0.05).

### 3.1. Echocardiographic Outcomes 

#### 3.1.1. Two-Dimensional Data Analysis and Systolic Function

The analysis by conventional methods showed that the hypertension group had larger left atrial diameter (39 versus 35 mm; *p* < 0.05) and volume (49.3 versus 33.9 mL; *p* < 0.05), LV concentric hypertrophy (13 versus 10 mm; *p* < 0.05), and lower LV systolic end volume (22 versus 25 mL; *p* < 0.05). Both groups had preserved LV ejection fraction, by Simpson's modified method. Taken together the body of data reveals homogeneity in systolic function (Table 2).

#### 3.1.2. Diastolic Function

In the hypertensive group, by pulsed Doppler we observed lower values of E wave velocity (61 versus 77 cm/s; *p* < 0.05), higher values of A wave velocity (92 versus 56 cm/s; *p* < 0.05), higher values of deceleration of mitral flow time (257 versus 194 ms), and higher values of isovolumetric relaxation time (123 versus 77 ms), that is, mild diastolic dysfunction. By tissular Doppler, in the hypertensive group we observed higher values of the E/E′ ratio (10.2 ± 3.1 versus 6.6 ± 1.1, *p* < 0.05) and higher levels of LVEDP estimated by Nagueh formula (16.2 ± 4.5 versus 11.3 ± 1.6 mm Hg), both consistent with diastolic dysfunction.

#### 3.1.3. Left Ventricle Global Longitudinal Deformation

With respect to the evaluation of ventricular mechanics by speckle tracking, the hypertension group showed lower longitudinal deformation than controls, −12.6 (−6.4 to −15.8) versus −16.9 (−13.1 to −22), respectively, (Table 3, Figures 1(d) and 1(e)), which confirms the diastolic dysfunction even when the LVEF is normal, that is, early left ventricular mechanical changes, detected by this echocardiographic technique.

### 3.2. Biochemical Outcomes

**Figure 2:**
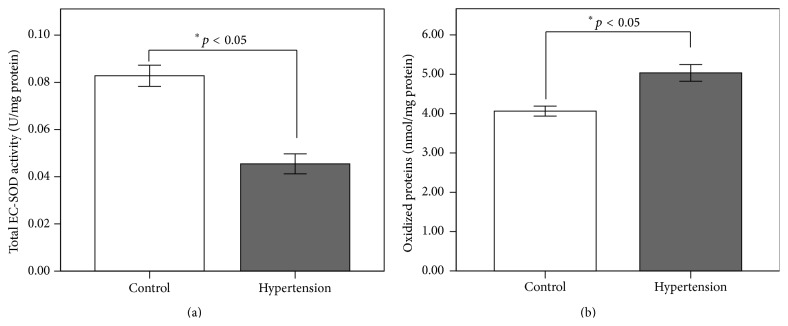
Total activity of EC-SOD (a) and levels of oxidized proteins (b) in control and hypertensive group. EC-SOD: extracellular superoxide dismutase. Student's *t*-test.

Biochemical evaluation revealed a total activity of EC-SOD lower in hypertensive patients when they were compared with the control group (0.045 ± 0.02 versus 0.082 ± 0.02 U/mg, *p* < 0.005); this can be explained by the chronic state of oxidative stress in these patients (Figure 2(a)). This is shown by the increased levels of oxidized protein, which were 24 percent higher in hypertensive patients versus controls (5.0350 ± 1.05 versus 4.0631 ± 0.63, *p* < 0.05) (Figure 2(b)).

### 3.3. Correlation between Oxidative Stress and Left Ventricular Mechanics by Speckle Tracking

**Figure 3:**
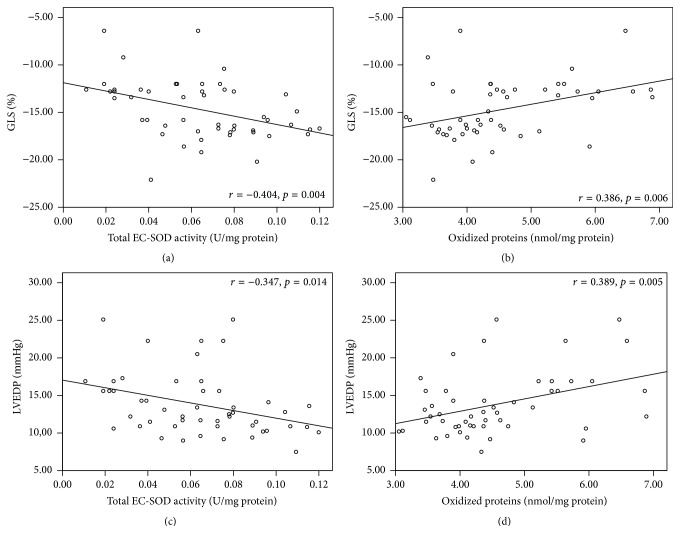
Relationship between global longitudinal strain and extracellular superoxide dismutase activity (a) and oxidized proteins (b). Relationship between left ventricular end diastolic pressure and total extracellular superoxide dismutase activity (c) and oxidized proteins (d). GLS: global longitudinal strain; EC-SOD: extracellular superoxide dismutase; LVEDP: left ventricular end diastolic pressure.

Figures 3(a) and 3(c) show a negative correlation of total superoxide dismutase activity with GLS (*r* = −0.404, *p* = 0.004) and LVEDP (*r* = −0.347, *p* = 0.014). Figures 3(b) and 3(d) show a positive correlation of the levels of oxidized proteins with GLS (*r* = 0.386, *p* = 0.006) and LVEDP (*r* = 0.389, *p* = 0.005).

Multivariate analysis revealed no significant differences adjusted for confusors variables (age, weight, body mass index, and antihypertensive treatment).

## 4. Discussion 

The oxidative stress state is characterized by an increase in the amount of reactive oxygen and nitrogen species that overwhelms the antioxidant defense systems of the body; this state is present in some chronic cardiovascular diseases such as hypertension.

All subjects in our study group were hypertensive and overweight, a common situation in emergent forms of the metabolic syndrome. However, due to the selection criteria, our group of patients did not fulfill the diagnosis of metabolic syndrome, and the multivariate analysis controlling for confusion variables did not show a statistically significant association.

The echocardiographic determination of atrial and LV dimensions in hypertensive patients clearly indicated that this group of patients had concentric LV hypertrophy, as the patients reported by Qu and collaborators [39], and that it was associated with the highest degree of diastolic dysfunction.

Wang et al. [32] demonstrated that, in patients with preserved systolic function and cardiac insufficiency data suggesting diastolic dysfunction, the values of GLS decreased compared with healthy controls, reporting an average of −12 ± 2%, whereas in our study we found a median GLS of −12.6% (−6.4 to −15.8). We found a positive correlation between oxidized proteins and GLS; that is, the greater the oxidative stress state (higher levels of oxidized proteins), the lower the myocardial longitudinal deformation (positive values of GLS) and the worse the systolic function, even with preserved LVEF. In addition, we confirmed a negative correlation between EC-SOD activity and GLS; that is, the lower the antioxidant defense state, the lower the myocardial deformation or the worse systolic function.

Sohn et al. [40] and Kasner et al. [35] reported that in diastolic dysfunction the E′ wave velocity is lower and the tissue Doppler A′ wave is longer, a situation that we observed in the hypertensive group.

The hypertensive group showed values of E/E′ > 10, a parameter that has been associated with high left ventricular end diastolic pressure and that, according to Sohn et al. [40], has shown a sensitivity of 75% and a specificity of 93% (the mean in our study was 16.2 ± 4.5 mm Hg).

On the basis of these findings, we argue that hypertensive patients have diastolic dysfunction, as documented by Doppler transmitral flow propagation velocity, color, mitral annular velocity, and higher LVEDP than controls.

Vaziri and Rodríguez-Iturbe [23] showed an increase in the production of reactive oxygen species in patients with essential, renovascular, and malignant hypertension, as well as preeclampsia; these findings were generally based on an increase in the plasma levels of reactive biomarkers of lipid peroxidation and oxidative stress. We found similar results in our study, reporting lower levels of EC-SOD activity in the hypertensive group.

Shizukuda et al. [41] reported a positive correlation (*r* = 0.32, *p* = 0.001) between peroxidized lipid concentrations and the degree of diastolic dysfunction as measured by the speed of the A wave. We found the same positive correlation between the levels of oxidized proteins and LVEDP; in other words, the greater the states of oxidative stress, the higher the LVEDP and also the worse the diastolic dysfunction, even with preserved LVEF.

Furthermore, Sagar et al. [42] reported that the decrease of antioxidant activity (superoxide dismutase) and decreased levels of scavengers of reactive oxygen species may contribute to oxidative stress state. Our results were consistent with a negative correlation between EC-SOD activity and LVEDP; that is, the higher values of LVEDP, the lower values of the antioxidant activity of EC-SOD.

## 5. Conclusion 

An increase in the levels of oxidized proteins and reduced antioxidant defenses (oxidative stress state) in essential hypertensive patients are correlated with a worse systolic function as measured by left ventricular global longitudinal systolic strain, which decreases even with normal left ventricular ejection fraction; they are also correlated with a reduced diastolic function as measured by an increase in left ventricular end diastolic pressure, both measured by echocardiography.

## Figures and Tables

**Table 1:** Anthropometric and demographic variables of both study groups.

Variables	Group I: control (*n* = 25)	Group II: hypertension (*n* = 25)
Age (years)	43.3 ± 8.04	43.8 ± 10.2
Women	10 (40)	10 (40)
Weight (kg)	68.9 ± 10.8^**∗**^	79.8 ± 12.4^**∗**^
Height (m)	1.65 ± 0.1	1.64 ± 0.1
Corporal mass index (kg/m^2^)	24.8 ± 1.0^**∗**^	29.4 ± 1.8^**∗**^
Systolic pressure (mm Hg)	100 (80–120)^†^	130 (90–150)^†^
Diastolic pressure (mm Hg)	60 (50–80)^†^	70 (60–90)^†^
Heart rate (bpm)	67 (56–75)	70 (60–90)

Values represent means ± SD, median (minimum-maximum) or *n* (percent). ^*∗*^ *p* < 0.05 (Student's *t*-test), ^†^ *p* < 0.05 (Mann-Whitney *U* test).

**Table 2:** Conventional echocardiographic measurements in both study groups.

Variables	Group I: control (*n* = 25)	Group II: hypertension (*n* = 25)
LA (mm)	35 (26–40)^†^	39 (33–48)^†^
RA (mm)	37 (32–41)	38 (35–49)
LVDD (mm)	44.3 ± 4.2	43.9 ± 2.9
LVSD (mm)	26 ± 3.6	25.6 ± 2.9
S (mm)	10 (8–11)^†^	13 (9–14)^†^
PW (mm)	10 (8–11)^†^	13 (9–14)^†^
RVDD (mm)	26 (23–30)^†^	28 (25–32)^†^
AoR (mm)	30 (25–35)	31 (27–35)
AoA (mm)	20 (17–22)	20 (17–21)
LVESV (mL)	25 (13–42)^*∗*^	22 (16–39)^*∗*^
LAV (mL)	32.12 ± 12.2^*∗*^	49.3 ± 10.4^*∗*^
RAV (mL)	33.9 ± 11.4^*∗*^	43.6 ± 13.1^*∗*^
PSP (mm Hg)	25.5 ± 3.3^*∗*^	29.2 ± 5.5^*∗*^
LVEF	0.70 ± 0.05	0.70 ± 0.06

Values represent means ± SD, median (minimum-maximum). LA: left atrium; RA: right atrium; LVDD: left ventricle diastolic diameter; LVSD: left ventricle systolic diameter; S: interventricular septum; PW: posterior wall; RVDD: right ventricle diastolic diameter; AoR: aortic root; AoA: aortic annulus; LVESV: left ventricle end systolic volume; LAV: left atrial volume; RAV: right atrial volume; PSP: pulmonary systolic pressure; LVEF: left ventricle ejection fraction. ^*∗*^ *p* < 0.05 (Student's *t*-test), ^†^ *p* < 0.05 (Mann-Whitney *U* test).

**Table 3:** Ventricular mechanics parameters measured by pulsed, M-color, and tissular Doppler and speckle tracking.

Variables	Group I: control (*n* = 25)	Group II: hypertension (*n* = 25)
E wave, cm/s	77 (49–118)^†^	61 (44–87)^†^
A wave, cm/s	56 (38–94)^†^	92 (67–103)^†^
E/A ratio	1.2 (1.0–2.2)^†^	0.7 (0.6–0.9)^†^
DT, ms	194 (127–246)^†^	257 (194–300)^†^
IRT, ms	77 (63–95)^†^	123 (103–140)^†^
E′ wave, cm/s	11.7 ± 1.9^*∗*^	6.3 ± 2.04^*∗*^
A′ wave, cm/s	8.3 ± 1.7^*∗*^	8.8 ± 1.0^*∗*^
E/E′ ratio	6.6 ± 1.1^*∗*^	10.2 ± 3.1^*∗*^
LVEDP, mm Hg	11.3 ± 1.6^*∗*^	16.2 ± 4.5^*∗*^
PV color, cm/s^2^	69 (52–99)^†^	40 (23–48)^†^
GLS, %	−16.9 (−13.1 to −22)^†^	−12.6 (−6.4 to −15.8)^†^

Values represent means ± SD, median (minimum-maximum). DT: E wave deceleration time; IRT: isovolumetric relaxation time; LVEDP: left ventricle end diastolic pressure (by Nagueh formula); PV color: transmitral color propagation velocity; GLS: left ventricle global longitudinal strain. ^*∗*^ *p* < 0.05 (Student's *t*-test), ^†^ *p* < 0.05 (Mann-Whitney *U* test).
